# Future-Oriented Experimental Characterization of 3D Printed and Conventional Elastomers Based on Their Swelling Behavior

**DOI:** 10.3390/polym13244402

**Published:** 2021-12-15

**Authors:** Klara Loos, Vivianne Marie Bruère, Benedikt Demmel, Yvonne Ilmberger, Alexander Lion, Michael Johlitz

**Affiliations:** 1 Institute of Mechanics, Bundeswehr University Munich, 85579 Neubiberg, Germany; vivianne.bruere@unibw.de (V.M.B.); benedikt.demmel@unibw.de (B.D.); yvonne.ilmberger@unibw.de (Y.I.); alexander.lion@unibw.de (A.L.); michael.johlitz@unibw.de (M.J.); 2 Bundeswehr Research Institute for Materials, Fuels and Lubricants, 85435 Erding, Germany

**Keywords:** swelling, absorption, infrared spectroscopy, mass spectrometry, gas chromatography, mechanical behavior, synthetic aviation fuels, 3D printed elastomers

## Abstract

The present study investigates different elastomers with regard to their behavior towards liquids such as moisture, fuels, or fuel components. First, four additively manufactured materials are examined in detail with respect to their swelling in the fuel component toluene as well as in water. The chemical nature of the materials is elucidated by means of infrared spectroscopy. The experimentally derived absorption curves of the materials in the liquids are described mathematically using Fick’s diffusion law. The mechanical behavior is determined by uniaxial tensile tests, which are evaluated on the basis of stress and strain at break. The results of the study allow for deriving valuable recommendations regarding the printing process and postprocessing. Second, this article investigates the swelling behavior of new as well as thermo-oxidatively aged elastomers in synthetic fuels. For this purpose, an analysis routine is presented using sorption experiments combined with gas chromatography and mass spectrometry and is thus capable of analyzing the swelling behavior multifacetted. The transition of elastomer constituents into the surrounding fuel at different aging and sorption times is determined precisely. The change in mechanical properties is quantified using density measurements, micro Shore A hardness measurements, and the parameters stress and strain at break from uniaxial tensile tests.

## 1. Introduction

Nowadays, polymers are used in a wide range of applications such as hoses, sealings, and membranes, where they come into contact with various surrounding liquids such as water [1,2], oils [3], acids [4], organic solvents [5], and fuels [6,7]. Polymers tend to swell, i.e., to absorb liquids, which is usually associated with an increase in volume. This leads not only to a change in physical parameters such as density and hardness but also to a change in mechanical properties.

The present paper investigates the swelling behavior of soft elastomers on the basis of various aspects. Firstly, attention is paid to the swelling behavior of additively manufactured soft polymers. The new additive manufacturing processes not only expand their field of application but also raise fundamental questions about the optimal choice of printing process parameters. The present study on the swelling behavior represents an enormously important step in the analysis of the additive manufacturing process. The printed end product differs greatly depending on whether a wet or dry filament was printed. Only the knowledge of how to set the correct printing parameters enables an optimal printing result.

The application of soft polymers in sealings and hoses, especially fuel-conveying hoses, motivates the further part of the presented study. Here, pristine as well as thermo-oxidatively aged elastomers are subjected to a study on their swelling behavior. The concrete question is ultimately the operational stability of fuel hoses in which new types of synthetic fuels are conveyed. The diffusion of fuel components into the polymer as well as the diffusion of polymer components into the surrounding liquid are investigated in detail in this study. The influence of swelling on the mechanical properties before and after aging is quantified in detail.

## 2. Swelling Behavior of Additively Manufactured Soft Polymers in Water and in the Fuel Component Toluene

Additive manufacturing (AM) gradually comes into focus for the fabrication of functional parts. With respect to soft polymers, the limitations of the AM technologies regarding both the type and the nature of the employed materials hinder the use of conventional elastomers. Instead, 3D printers may operate with other types of existing materials as well as with newly developed ones. AM parts show significantly different mechanical properties compared to the same parts produced with conventionally produced elastomers, e.g., natural rubber. Therefore, generating knowledge on this subject is a fundamental step toward understanding the mechanical behavior of these materials from 3D printing processes and the feasibility of their applications. This contribution aims to give a brief overview of the additive manufacturing of elastomers and to show the influence of liquid media, such as moisture and fuels, on the printing process as well as on the subsequent application. Regarding their chemical composition, the investigated materials are initially analyzed, while the focus of this work lies on swelling tests with water and toluene followed by uniaxial tensile tests.

### 2.1. Elastomers in the AM Scenario

Popularly known as 3D Printing, the origins of AM are associated with the prototyping industry. Nevertheless, it has become a more and more interesting alternative for the production of parts with technical applications in addition to conventional manufacturing methods, such as machining and injection molding. The layer-wise process of joining materials to form parts from 3D model data allows a less wasteful, on-site manufacturing along with the exemption of individual tooling and reduction of postprocessing [8,9,10]. AM is particularly advantageous for the small-scale fabrication of small and complex custom-made parts. As considerable progress is constantly achieved, not only on the technologies themselves but also on the employed materials, a wider range of products and applications is increasingly allowed.

In this context, we can find the elastomers. Several current technologies are able to print parts based on rubber-like materials. It is important to point out, though, that every AM technology demands a specific type and nature of material, which generally does not allow the use of traditional, vulcanized rubber. One of the main reasons is that the vulcanization process cannot easily be transferred to AM. Some technologies, however, allow the use of conventional liquid silicone rubber. Alternatively, thermoplastic elastomers (TPEs) and photopolymers are employed in the printing of soft, elastic components.

One of the downsides associated with those alternative materials is typically the still inferior mechanical behavior [11,12] in terms of operational performance and service life compared to conventional materials. The combination of large deformations with complete recovery at a required demanding stress along with long-term stability is not always fully feasible, for instance. Nevertheless, the continuous development in material science changes the everyday scenario; materials are improved, and new ones are created with the purpose of enhancing the mechanical properties. At the moment, research on such materials is of considerable significance in order to generate both knowledge and a better understanding of their behavior from an engineering point of view. In this way, it is possible to be aware of the properties of current materials for AM of elastomeric parts and help in the optimization of the related printing processes, so that they can also be of use in functional parts instead of being limited to prototyping and demonstration.

**Figure 1:**
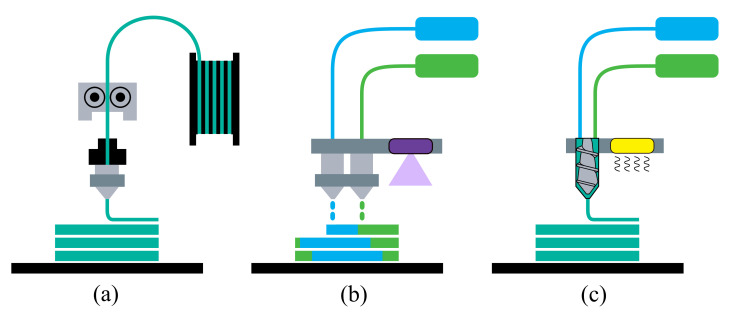
AM processes for (**a**) fused filament fabrication (FFF), (**b**) PolyJet, and (**c**) liquid additive manufacturing (LAM).

Some of the today’s AM technologies that are able to process elastic materials are fused filament fabrication (FFF), PolyJet, and liquid additive manufacturing (LAM), which are explored in this section and sketched in Figure 1. FFF—also denoted by the trademarked name fused deposition modeling (FDM) by the company Stratasys^®^—is one of the most popular, low-cost AM processes. In this technology, the material in filament form is fed through a heated nozzle up to melting and pushed in it by a motor, characterizing the deposition by means of extrusion, after which the material cools down and solidifies into the desired geometry. Since this process demands the use of thermoplastics, TPEs are the choice for the printing of elastomeric parts. PolyJet is a material jetting process, working similar to a standard 2D inkjet printer and equipped with multiple nozzles. These nozzles deposit droplets of photopolymers that are then cured by exposure to UV light. The LAM technology is based upon the extrusion of a liquid or high-viscosity material onto a build plate, where the two-part component materials are mixed in a screw-like manner right before the deposition. Then, heat is provided to carry out the crosslinking by thermal energy.

Overall, AM is a relatively young manufacturing process in the elastomeric field that has a lot to be explored, although very promising. Both the market and the industry can benefit themselves not only with the fabrication of new parts on demand, reducing warehousing costs, but also with the faster replacement of damaged components, particularly those discontinued by original manufacturers. Despite being more cost-effective for small batch sizes, the evolution of the technologies and materials helps in gradually enabling the use of AM for mass production at a competitive market price in comparison with the traditional fabrication methods. Nevertheless, ensuring the quality of the final printed part is imperative to establish AM in the field of rapid manufacturing.

### 2.2. Overview of the Investigated 3D Printed Elastomers

The following subsections present chemical and mechanical investigations for 3D printed elastomers of three main types of currently available materials: liquid silicone rubber (LSR), thermoplastic polyurethane (TPU), and photopolymers. There are four printable materials of interest for this research. SILASTIC™LC 3335, supplied by Dow^®^ (Midland, MI, USA) is a two-component viscous silicone with a hardness of 50 Shore A and printed with the LAM process in an innovatiQ’s LiQ 320 printer (Feldkirchen, Germany). The second material is Filaflex 70 A, a TPU from company Recreus (Elda, Spain), with a hardness of 70 Shore A.Using this material, parts can be manufactured by a FFF process, which was performed in an Original Prusa i3 MK3S+ 3D printer (Prag, Czech Republic) for this work. The third and fourth materials can be processed with the PolyJet technology. They consist of the same type of photopolymer of resinous material with the name TangoBlackPlus combined with VeroClear (referred here simply as Tango+), supplied by Stratasys^®^ (Rechovot, Israel), but vary in hardness with 70 and 50 Shore A, respectively. For those, a Stratasys^®^ Object500 Connex3 printer (Material Jetting—MJ) was used. Table 1 shows some of the mechanical properties of these polymers according to the supply companies. All samples were printed with a 100% infill. A 90°-line orientation was used for LSR and TPU, and drops were applied for Tango+.

A chemical characterization was performed using infrared (IR) spectroscopy. The changes in weight caused by moisture absorption and exposure to toluene were investigated by means of water uptake and solvent absorption tests. At last, tensile tests allowed comparisons of the mechanical properties of the different elastomer samples before and after the water uptake tests. For the solvent absorption tests, however, no tensile properties were able to be evaluated since the intense toluene swelling damaged the samples.

### 2.3. Chemical Composition Analysis via IR Spectroscopy

**Figure 2:**
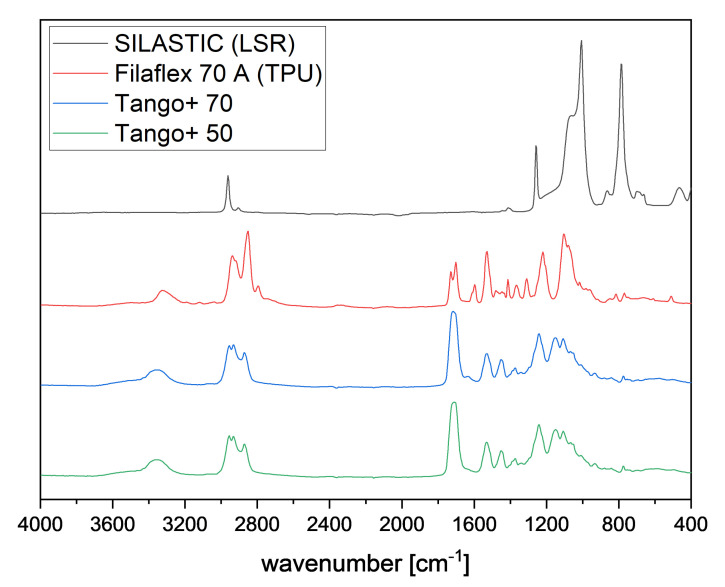
FTIR spectra for SILASTIC, Filaflex 70 A, Tango+ 70, and Tango+ 50.

To characterize the polymers, Fourier Transform infrared spectrometer (FTIR) spectra were recorded with an attenuated total reflectance (ATR) unit. The measuring device was a Bruker Tensor 27 with Platinum ATR and 32 scans were taken per sample in the range between 400 and 4000 ${\mathrm{cm}}^{-1}$ with a resolution of 4 ${\mathrm{cm}}^{-1}$. Figure 2 shows the IR spectra of the four above mentioned polymers.

The absorption bands for the silicone SILASTIC at 1065 and at 1007 ${\mathrm{cm}}^{-1}$ are assigned to the Si-O-Si backbone. The bending band of the Si-CH${}_{3}$ is found at 1260 ${\mathrm{cm}}^{-1}$, and the coupling stretching of Si-C and rocking band of -CH${}_{3}$ is assigned at 788 ${\mathrm{cm}}^{-1}$. The absorption band at 2962 ${\mathrm{cm}}^{-1}$ belongs to the stretching vibration of CH${}_{3}$. These determined bands can be assigned to the specific bands of silicones [13,14].

The TPU Filaflex 70 A shows pronounced absorption bands at 2939 and at 2851 ${\mathrm{cm}}^{-1}$, which are the characteristic bands of the aliphatic C-H asymmetric and symmetric stretching. In the range of 1105 ${\mathrm{cm}}^{-1}$, aliphatic ether groups are displayed. The spectrum also exhibits bands at 1731 and at 1702 ${\mathrm{cm}}^{-1}$ due to free and hydrogen-bonded urethanic C=O stretching. The band based on the N-H stretching vibration of the urethane amide was observed at 3323 ${\mathrm{cm}}^{-1}$. The sharp band at 1530 ${\mathrm{cm}}^{-1}$ corresponds to the C≡N stretching and N-H bending. The band at 1220 ${\mathrm{cm}}^{-1}$ is in accordance with the stretching vibration of urethanic -C-(C=O)-O-. These observations indicate that the TPU used in this work is a polyether polyurethane [13].

As the spectra of the Tango+ 70 and the Tango+ 50 are very similar, they are considered together. Just like the TPU, Tango+ also shows the specific bands at 1530 and at 1240 ${\mathrm{cm}}^{-1}$ for urethane content. The band at 1710 ${\mathrm{cm}}^{-1}$ indicates the presence of both acrylate and urethane. The characteristic bands of aliphatic C-H asymmetric and symmetric stretching are in the range from 2956 to 2873 ${\mathrm{cm}}^{-1}$. This indicates that the Tango+ material used in this study is a polyurethane acrylate [13,15].

### 2.4. Absorption Experiments with Toluene and Water

The geometries of tensile test specimens according to the Standard DIN 53504:2017-03-S2 [16] for SILASTIC, Filaflex 70 A and Tango+ were used for the absorption experiments carried out according to the Standard DIN ISO 1817:2016-11 [17] with the solvent toluene and water. Prior to the absorption experiments, the test specimens were conditioned under a standard climate (23 °C and 50% humidity), and the initial masses were determined. The mass measurements were made using a Sartorius Secura^®^ 225D-1S precision balance with a range of 120 g and a resolution of 0.0001 g. Each specimen was then stored in a screw glass containing 50 mL of toluene or water, respectively. After defined periods of time, the sample was taken out, dried with a lint-free paper, and weighed. After this, it was stored again in the solvent. To minimize the evaporation of the fluid out of the specimen, special care was taken by an immediate weight measurement. Three specimens were used for each material, and the results of the measurements were expressed in terms of the mean average values and standard deviations

#### 2.4.1. Absorption Test with Toluene as Solvent

**Figure 3:**
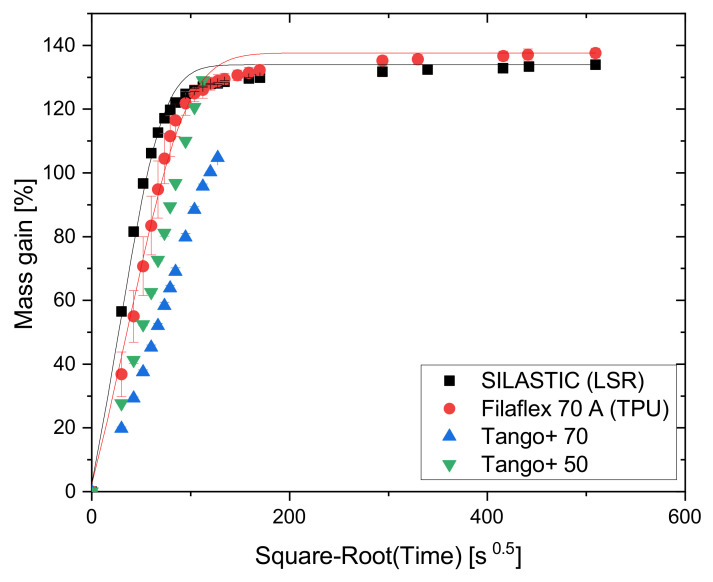
Absorption curves of the four AM polymers in toluene.

Figure 3 shows the amount of toluene absorbed in relation to the initial mass in terms of the percentage mass gain as a function of time. To account for Fickian diffusion, the sorption times on the abscissa are reported as the square root of time t${}^{0.5}$. In the beginning, an almost linear mass absorption takes place until the diffusion process slows down, and finally, in the equilibrium state, the mass does not change anymore. The results indicate a pronounced swelling of about 135% for SILASTIC and Filaflex 70 A. The saturation was reached after 24 h (≈294 s${}^{0.5}$). The procedure applied to SILASTIC and Filaflex 70 A was not suitable for the Tango+ materials. Tango+ 50 and Tango+ 70 were so heavily swollen by toluene that they could not hold their shape and broke after 3.5 h (≈112 s${}^{0.5}$) and 4.5 h (≈127 s${}^{0.5}$), respectively. This is due to their drop-like construction. Toluene accumulates at the boundary surface of the droplets. This softens the bonding between the drops. Since a little pressure already let the bonds break and, thus, the integrity of the samples was jeopardized, the measurement was aborted.

The solid lines in Figure 3 show the theoretical sorption curves normalized to the initial mass calculated by iteratively fitting Equation (1) to the available measurement data. This formula, solved with the method of least squares, describes an ideal model of absorption in accordance with Fick’s Law [18], where $M_{t}$ and $M_{\infty }$ represent the mass of the absorbed solvent at the time *t* and in the equilibrium state, respectively, *D* is the diffusion coefficient, *h* is the sample thickness, and *n* is the summation index.

(1) $$\begin{array}{c} \frac{M_{t}}{M_{\infty }}=1-\frac{8}{\pi^{2}}\sum_{n=0}^{\infty }\left(\frac{1}{{\left(2n+1\right)}^{2}}\exp\left(\frac{-{\left(2n+1\right)}^{2}\;\pi^{2}\;D\;t}{h^{2}}\right)\right) \end{array}$$

Figure 3 shows that toluene has different effects on the three polymer types used, due to their different chemical structure. It can also be seen that SILASTIC and Filaflex 70 A comply with Fick’s Law. As the complete testing was not possible for the Tango+ materials, no statements can be made in this regard. However, attention must also be paid to the printing parameters as they also influence the swelling behavior. Changing the infill pattern, the degree of infill, or the number of perimeters leads to different absorption properties.

#### 2.4.2. Absorption Test with Water

**Figure 4:**
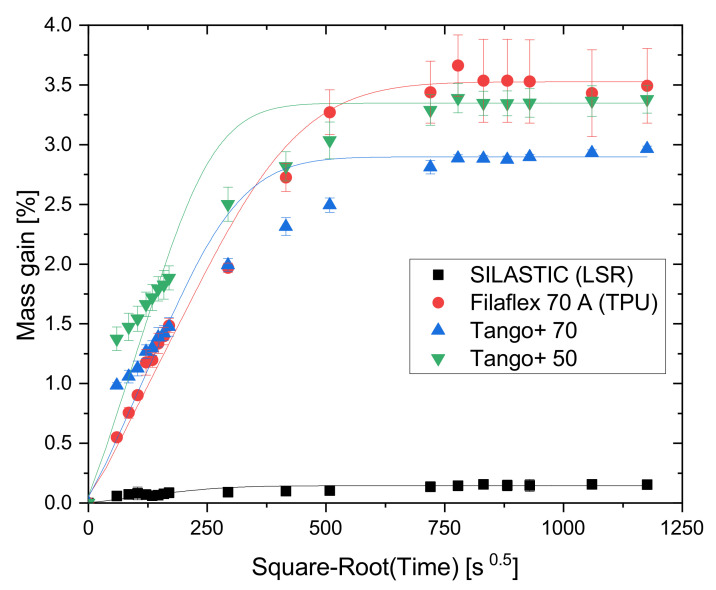
Curves for water absorption evolution.

The hygroscopic nature of the four chosen elastomeric materials was analyzed in a Water uptake test. The results are plotted along with the fitted curves based on Fick’s Law from Equation (1) in Figure 4. It can be observed that the photopolymers and the TPU are particularly hygroscopic, while the LSR samples exhibit a much lower water uptake less than 10% of the others. It can also be seen that the TPU follows Fick’s Law. No statement can be made about the behavior of the SILASTIC material, as the individual measured values scatter strongly in the initial range. In comparison with TPU, the two more hydrophilic photopolymers Tango+ show a different absorption behavior: the polymer chains form more hydrogen bonds with the water molecules and free unbound water molecules in the voids between the chains show Fickian diffusion behavior [19].

**Figure 5:**
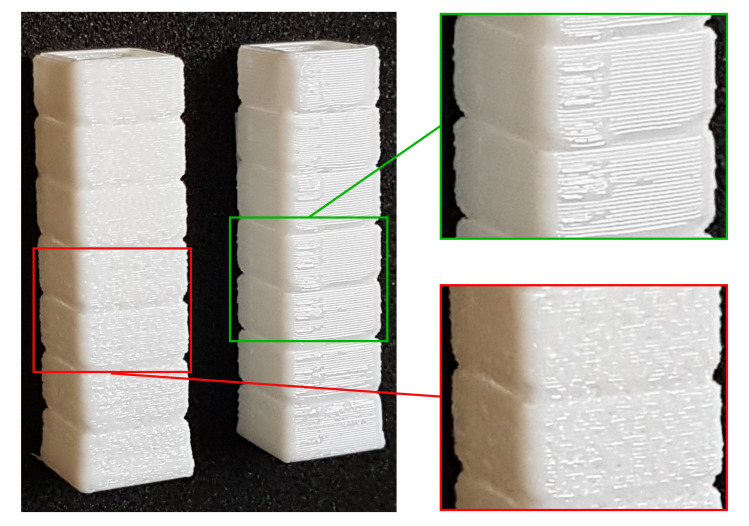
Influence of material condition prior to FFF printing on final geometry for a moist (red) and a dried (green) Filaflex 70 A filament.

A considerable part of the absorption for all materials occurs during the first hours of the experiment; in the first 24 h, more than half of the saturation is already achieved. This evidences the importance of the environment in functional applications. While water does not play a major role in the conventional rubber-like material LSR, the same cannot be affirmed for the photopolymers and the TPU. In fact, it is already known that TPEs in general are hygroscopic, which should be taken into consideration, notably before and during the FFF printing process. Filaments with increased moisture levels lead to extrusion failures and the generation of voids on the streaming, caused by steam formation on the heated nozzle [20]. Moreover, depending on the printing environment and the exposure time, the filament spools in use may constantly absorb air moisture. Figure 5 visualizes the consequences of printing with a moist filament, which gives a surface that is more opaque and with less uniform strand deposition than for geometries created with a dry filament. The excess of material on the left-hand side of the printing with a dry filament (green) is due to the accumulation of random start and end points of the deposition. Therefore, the material should be properly stored not only before/after printing but also while the object is being constructed to ensure good printing quality.

### 2.5. Mechanical Behavior Analysis via Uniaxial Tensile Tests

**Figure 6:**
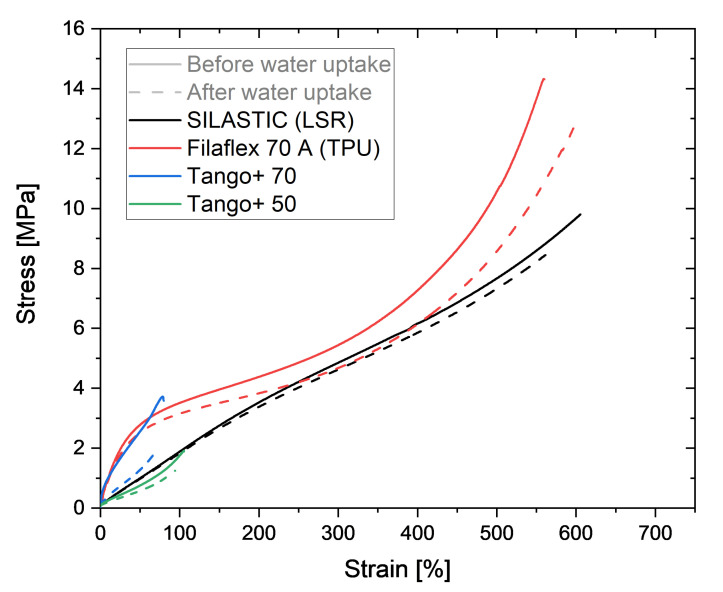
Stress–strain curves until break for the four different AM polymers before and after water absorption.

The effect of moisture on the mechanical behavior of the printed samples was evaluated by tensile tests on a Zwick Roell 1445 universal testing machine with a force sensor of 500 N and an optical extension sensor ProLine lightXtens 2-1000 at ambient temperature, a preload of 0.1 MPa and a strain rate of 200 mm min${}^{-1}$, according to the Standard DIN 53504:2017-03 [16]. The stress–strain curves of the samples before and after the water absorption test in the saturation state were measured. The average results of three samples for each material and condition are displayed in Figure 6. The values for the ultimate stress and the elongation at break can be found in Table 2. Due to the pronounced swelling and the resulting low strength or breakage of the specimens, the tensile test after toluene absorption was omitted.

The curves show a decrease in stiffness and ultimate stress for the photopolymers Tango+, which is more pronounced for Tango+ 70 than for Tango+ 50. Indeed, there is a decrease of 50% on the tensile strength for Tango+ 70, although the elongation at break has a change of only 12%. For Tango+ 50, the tensile stress is lower by 31%, while the elongation at break decreases by 8%. It could be inferred that the higher the hardness for Tango+, the greater the effect of water saturation on the ultimate strength. Further investigations could corroborate that hypothesis.

Filaflex 70 A also shows that moisture influences its tensile properties. While the stress decreases by only 8%, the elongation at break increases by 10%. This is due to the hydrophilic character of the polymer chains. The water surrounds the chains like a lubricant and allows them to slide off each other more easily when pulled. This results in a higher elongation at break.

The LSR elastomer does not show any indication of alterations in the behavior before water uptake and after water saturation. The deviations in the stress–strain curves can be attributed to the variability of the individual samples in general. In fact, the small influence on the tensile results could already be expected due to the low saturation content for the LSR.

### 2.6. Intermediate Conclusions and Remarks

The present work explores the effects of absorption of toluene and water on three types of available 3D printed elastomers: liquid silicone rubber, thermoplastic elastomer, and photopolymer. Moreover, chemical analyses via IR Spectroscopy and mechanical analyses via uniaxial tensile tests complement the investigations. The results reveal the intense swelling response of all materials when exposed to toluene, while more diverse outcomes are displayed for water absorption. Furthermore, not only the chemical composition of the materials but also the nature of the printing technology have a direct impact on both the absorption behavior and the mechanical performance.

It should be considered that the AM processes are subject to variations not only on the tensile results but also on mechanical testing in general, according to the printing conditions as well as the postprocessing. The LSR and the photopolymers, for example, can go through a postcuring stage, increasing their stiffness; FFF printers allow the user to change the path and the direction of the material deposition. AM can lead to greater process deviations than the conventional and established manufacturing techniques. For that reason, 3D printing optimization is one of the current challenges in order to deliver functional parts in similar and reliable performance.

**Figure 7:**
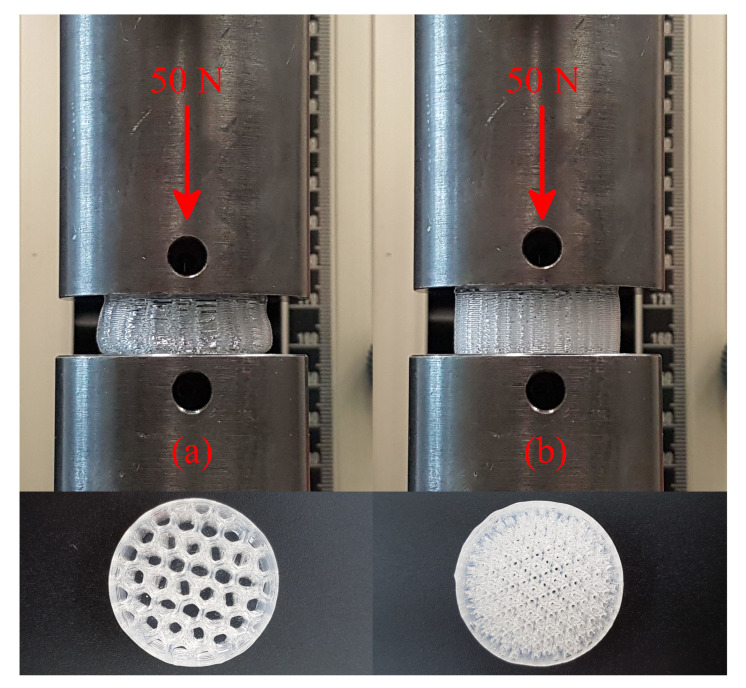
Compression of SILASTIC cylinders printed with infill percentages of (**a**) 25% and (**b**) 50%.

In addition, new materials emerge as a way to improve the quality of the printed parts. Nevertheless, since AM is a flexible process, the printing of the geometry can always be adapted to exhibit specific properties. As an example, we find the percentage of infill influencing on the compression of the part. For elastomeric materials with higher hardness, it is possible to increase the compressibility by decreasing the infill percentage. Figure 7 exemplifies that two geometrically identical cylinders printed with the same material (SILASTIC, 50 Shore A) in different infill percentages of 25% and 50% exhibit distinct performances when compressed with a force of 50 N.

The investigations presented here are a significant step toward the study of the feasibility of 3D printed elastomeric parts for technical applications. Knowledge on water uptake and toluene absorption, for instance, is beneficial when deciding the printing material for a defined geometry in a specific application. It can be noted that special attention must be taken when using the studied photopolymers and TPU in water applications, as moisture absorption cannot be neglected. Silicone could be better suited for moist environments, on the other hand. The toluene absorption tests show the relevance on applications involving contact with fuels, in which the silicone and the TPU revealed an intense swelling behavior, while the photopolymers were not able to withstand the solvent. With such a piece of information, the process of selecting the material and manufacturing options in AM becomes accessible in future works.

## 3. Swelling Behavior of Pristine and Thermo-Oxidatively Aged Elastomers in Synthetic Fuel

Synthetic fuels are attracting considerable interest as alternative energy sources in the aviation sector [21,22]. The main advantages are their high volumetric energy density and renewable feedstocks. One of the crucial topics is a possibly different or adverse interaction between the fuels and construction materials in the aircraft, in comparison to conventional kerosene [23]. Especially soft materials, such as elastomers in sealings, tank hoses, and linings, respond distinctly to fuel contact by swelling, extraction of plasticizers [24], and a change in mechanical properties. So far, most studies focus on the interaction of fuels with pristine elastomers. Graham et al. [6] investigated the influence of selected aromatics blended in a synthetic jet fuel on the volume swell by correlation with partition coefficients determined with gas chromatography/mass spectrometry (GC/MS). Blivernitz et al. [7] developed a method for the simultaneous and time-resolved quantification of sorption and extraction processes of individual model fuel components and elastomer additives via GC/MS assisted sorption experiments. Acrylonitrile-butadiene-rubber (NBR) is a material typically used for applications with nonpolar fuels and is part of many aircraft seals. With long aircraft service times, especially in the military sector, the elastomer properties change due to aging [25]. This contribution presents an advanced analytical method to study diffusion processes of NBR in contact with liquid media after thermo-oxidative aging. The focus lies on an in-depth understanding of the diffusion processes of single substances and substance classes in model and real fuels by gas chromatography/mass spectrometry combined with mechanical testing.

### 3.1. Kerosene and Synthetic Aviation Fuels

Up to now, conventional kerosene, such as Jet A-1, made from non-renewable crude oil, is the main energy source in the aviation sector [22]. To tackle the environmental crisis by reducing the CO_2_ emissions and saving resources, innovative solutions are required. Although other technologies such as hydrogen or fuel-cell-powered [26] and battery-electric aircrafts [27] are considered and in development, synthetic aviation fuels are the most important alternative energy source at the moment [28]. The advantages of liquid fuels are their high gravimetric and volumetric energy density and the potential to use existing aircraft and infrastructure. Furthermore, the competing technologies need further improvements to become competitive, but finally a mix of technologies must be considered and tailored to the respective applications. Liquid aviation fuels in a chemical view are composed of different hydrocarbon classes: linear alkanes, branched isoalkanes, cycloalkanes, and aromatics [29]. The fuel properties are highly dependent on the chemical composition and the distribution of the hydrocarbons. While conventional kerosene contains aromatic substances, certain types of the synthetic fuels do not. Because the aromatic hydrocarbons increase the swelling for a lot of elastomers, there are mainly three strategies to meet the specified range of 8–25 vol% aromatics in fuels: First, blend aromatic-free fuels with petroleum-derived kerosene [30,31]; second, add aromatic additives; or, third, design processes to produce so-called drop-in fuels, which are synthetic fuels with aromatics and capable of replacing kerosene equally. All relevant parameters for conventional kerosene are specified in the Standard ASTM D1655 [32] and Defence Standard 91-091 [33]. To stay abreast of the current developments regarding synthetic fuels from various renewable feedstock, the Standard ASTM D7566 [34] and Annex D of Defence Standard 91-091 [33] were established. The specification process for new fuels is strictly regulated [35], because of the high safety standards in the aviation sector.

### 3.2. Testing the Interactions of Elastomers with Synthetic Fuels

However, some fuels are already certified and tested thoroughly. Apart from fulfilling the fuel specifications, the ongoing challenge is material compatibility, since liquid fuels are in contact with soft elastomeric materials such as sealings, tank hoses, and linings. The next decade is likely to witness a considerable rise in the demand for synthetic fuels attributable to the ambitious goals of different advocacy groups. The aim of the International Air Transport Association (IATA) is to reduce global aviation-caused CO${}_{2}$ emissions by 50% relative to the level of 2005 until 2050 [36]. One intermediate goal in Germany is to achieve a 2% synthetic fuel stake by 2025 [37]. On account of its multidisciplinary nature, there is an immense potential to investigate this topic experimentally. For instance, fuel properties, the chemical and physical aging of elastomers with fuel contact [38], and swelling and diffusion phenomena [3] may be investigated. The swelling of the elastomer can also be simulated using constitutive models [39]. On a bigger scale, fuel-burning tests [40] or ultimately flight tests are conducted. Regarding material compatibility, mechanical studies are essential to investigate the tightness of seals [41], leakage due to shrinkage, when refueling or mechanical properties in dependence of the swelling status [42]. Whereas previous work is often limited to the interactions of pristine elastomer with fuels or fuel-like substances, the current study aims to estimate long-term effects by conducting experiments with pre-aged elastomers.

### 3.3. Experimental Part

Carbon black filled and stabilized NBR with a ready-to-use formulation containing 18 wt% acrylonitrile is used here, cf. Table 3. It contains further carbon black N550 (60 phr), the plasticizer DEHP (20 phr), the antioxidant 6PPD (2 phr), sulfur (2 phr) and vulcanizing agents, and the composition are the same as in a previous study [7]. Dumbbell-shaped specimens (S2) with a thickness of 1.2 mm are aged thermo-oxidatively, in a Binder ED56 oven with natural convection at 120 °C for three and seven days, without mechanical load. Applied Research Associates (ARA) produces the synthetic jet drop-in fuel ReadiJet™ used here. Chemically, it is a complex mixture of hydrocarbons similar to the conventional kerosene Jet A-1 with an aromatics content of 21.2 vol% measured according to Standard ASTM D1319-20a [43] and a density of 0.823 g cm${}^{-3}$ [29,30] and is used as the immersion fluid.

#### 3.3.1. Testing Procedure

**Figure 8:**
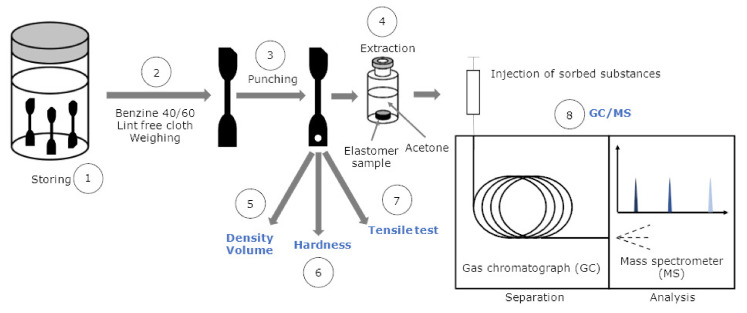
Versatile testing procedure of pristine and aged elastomer samples in contact with synthetic fuel.

Each sample was tested as a threefold measurement. Samples are pristine or aged for 3 d as well as for 7 d at 120 °C. For clear identification purposes, the specimens were marked clearly with cut-off edges. At first the samples were weighed, and their thickness was measured. The samples were then immersed in ReadiJet and taken out after distinct sorption times of 30 min (=42 s${}^{0.5}$), 2, 6, 24, 48, and 72 h and one week (=778 s${}^{0.5}$) (see Figure 8 (1)). To account for Fickian diffusion, the sorption times on the abscissa are reported as the square root of time t${}^{0.5}$. In other elastomer–fuel combinations, longer or shorter storage times in the fuel are possibly needed, depending on their compatibility. After the samples were taken out, they were dipped quickly in low boiling benzine 40/60 to remove adhering fuel, dried with a lint-free paper cloth, and then weighed again (2) to determine the mass change. Subsequently for GC/MS analysis, small pieces of ≈8 mg were punched out (3) and stored (4) in 2 mL GC vials that were filled with 1 mL acetone previously to extract the absorbed substances. To transfer the sample as quickly as possible into the vial, a funnel fixed to a stand was used. In the following step, the density using the Archimedes principle (5), micro Shore A hardness (6), according to Standard ISO 7619-1:2010 [44] and tensile properties such as stress at break and elongation at break according to Standard DIN 53504:2017-03 [16] were determined (7). In this case, the solid–liquid extraction of the absorbed substances in acetone was complete in two days. The acetone extract is then analyzed with GC/MS (8) described in the next subsection. To reduce the evaporation of the fuel, it is advisable to conduct the experiments immediately. For short times, the specimens may be covered in plastic bags to bridge transport times. The weighing was performed at the Sartorius precision balance mentioned in Section 2.4. Volume and density were determined using the Archimedes principle with the kit VF 4601 from Sartorius. The hardness was measured with a digi test II, by bareiss^®^ equipped with a micro Shore A hardness test head. Tensile tests were conducted with the universal testing machine used in Section 2.5. The samples were not preconditioned under cyclic load to preserve the secondary network, formed by aging-induced oxidative crosslinking. The strain rate is 0.167 s${}^{-1}$ = 200 mm min${}^{-1}$, respectively.

#### 3.3.2. Gas Chromatography/Mass Spectrometry (GC/MS)

**Figure 9:**
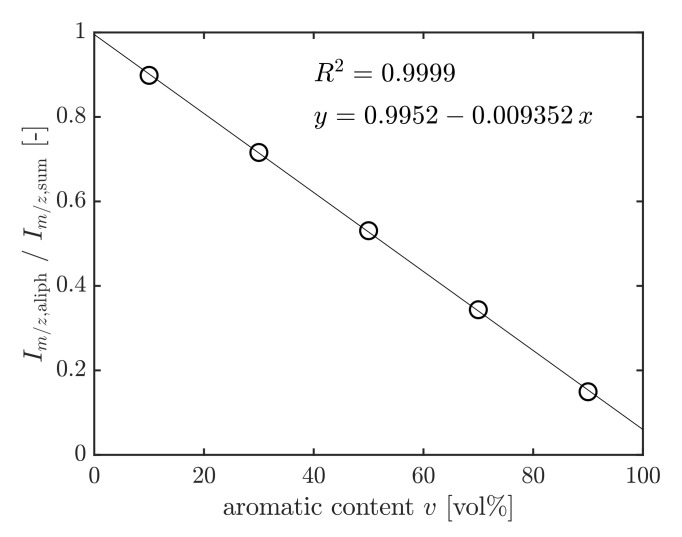
Calibration graph.

An Agilent 7890A gas chromatograph coupled with an Agilent 5975 MSD mass spectrometer was used to perform the GC/MS analysis. The column was a 30 m DB-5MS (0.25 mm inner diameter, 0.25 µm film thickness). The GC oven was heated with 50 K min${}^{-1}$ from 50 to 320 °C and held for 5 min at 320 °C. By reason of the chemical diversity of fuels, a defined surrogate fuel was prepared and used to calibrate and quantify the aromatics content of unknown samples. First, a mixture (AroMix) with a distribution of aromatic hydrocarbons, comparable to JP-8 jet fuel, was prepared by mixing 25:53:22 vol% of the aromatic liquids 100, 150, and 200 provided by Exxon [45,46]. An aromatic-free coal-to-liquid (CtL) fuel from Sasol was then blended with 10, 30, 50, 70, and 90 vol% of AroMix to yield the surrogate fuels, which were used as calibration standards for determining the aromatics content. Definite mass-to-charge ratios m/z of mass spectra signals were characteristic of aliphatic or aromatic hydrocarbons. Their signal intensities are referred to as $I_{m/z,\mathrm{aliph}}$ and $I_{m/z,\mathrm{aro}}$. After the GC/MS analysis of the standards, the respective characteristic intensities were added up in the retention time range of the fuel. Then, the sum of aliphatic signals $I_{m/z,\mathrm{aliph}}$ was set in relation to the sum of aromatic and aliphatic hydrocarbons $I_{m/z,\mathrm{sum}}$ according to Equation (2). The ratio $I_{m/z,\mathrm{aliph}}/I_{m/z,\mathrm{sum}}$ was plotted versus the aromatics content *v* of the standard solutions in vol% to yield the calibration graph [46] (see Figure 9):

(2) $$\begin{array}{c} \frac{I_{m/z,\mathrm{aliph}}}{I_{m/z,\mathrm{sum}}}=\frac{\sum_{i=1}^{9}I_{m/z,\mathrm{aliph},i}}{\sum_{i=1}^{9}I_{m/z,\mathrm{aliph},i}+\sum_{i=1}^{7}I_{m/z,\mathrm{aro},i}}\\ m/z,\mathrm{aliph}=41,55,57,69,71,83,85,97,99\;n=9\\ m/z,\mathrm{aro}=91,105,115,119,120,128,142\;n=7 \end{array}$$

It is important that the concentration of the standards is in the same range as the samples. Furthermore, it is beneficial to analyze independent samples with known aromatics content additionally to verify the calibration. The additives DEHP and 6PPD were quantified with external standards of the pure substances in acetone in appropriate concentrations. The known concentrations were plotted versus the peak areas of the additives in the chromatogram to obtain the calibration graph and function [47].

#### 3.3.3. Results

**Figure 10:**
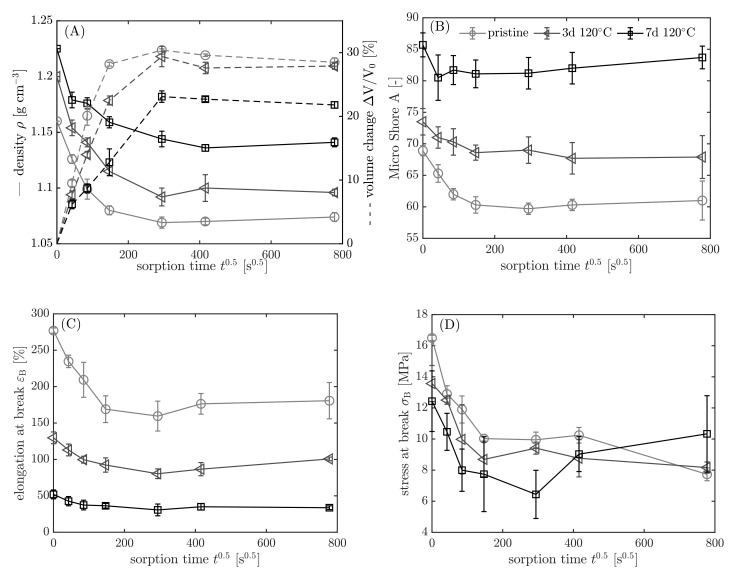
(**A**) Density and calculated volume change, (**B**) micro Shore A hardness, (**C**) elongation at break, and (**D**) stress at break according to the procedure in Figure 8 (step 5–7) in dependency on sorption time $t^{0.5}$. Samples: light-grey circles ◯: pristine; grey triangles ◁: 3 d 120 °C; black squares ☐: 7 d 120 °C.

In the following section, the results are shown in dependency on the aging history and sorption time. Pristine samples are represented by light grey (◯), aged samples for 3 days at 120 °C by dark grey (◁), and 7 days at 120 °C by black color (☐). In Figure 10, the results from density measurements and the calculated volume change (A), micro Shore A hardness (B), elongation at break (C), and the stress at break (D) according to the procedure in Figure 8 are shown.

Before the samples are immersed ($t^{0.5}=0\;s^{0.5}$), the impact of the aging is evident. The material becomes harder, denser, and thus less resilient with longer aging time. The density increases 5.6% from 1.16 to 1.225 g cm${}^{-3}$ after aging for 7 days at 120 °C compared to the pristine sample. Causes for that are volume shrinking due to the loss of additives, and a mass gain, because of oxygen binding to the elastomer. The mass gain is detectable by weighing when elastomers without volatile additives are thermo-oxidatively aged at elevated temperatures. With proceeding sorption time (t>0 s${}^{0.5}$), the density decreases, whereas the volume increases due to fuel uptake until the swelling equilibrium is reached. The volume increase is less pronounced for the aged samples, because the polymer chains of NBR form a secondary network, when they react with oxygen at elevated temperatures due to crosslinking [48]. The increased crosslink density restricts the intake of fuel into the elastomer. In the swelling equilibrium ($t^{0.5}=778$ s${}^{0.5}$), the volume increase is 23.5% smaller for the aged sample (7 d 120 °C) compared to the pristine sample. In addition, the micro Shore A hardness, the elongation at break, and the stress at break decrease with progressing sorption time due to the uptake of fuel and the extraction of the additives. For the aged samples, the change in the properties is smaller because less fuel is absorbed by the elastomer.

**Figure 11:**
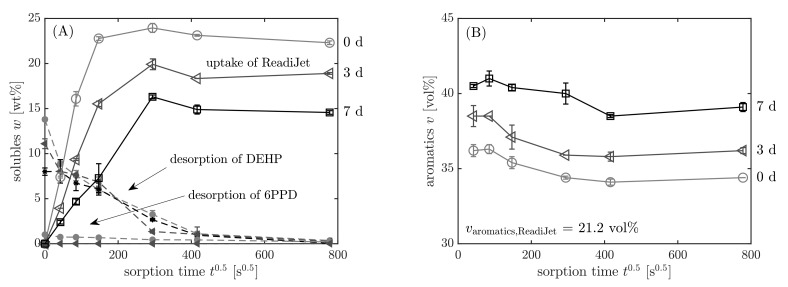
(**A**) Uptake of ReadiJet and desorption of DEHP and 6PPD, (**B**) aromatics content according to the procedure in Figure 8 (step 8) in dependency on sorption time $t^{0.5}$. Samples: light-grey circles ◯: pristine; grey triangles ◁: 3 d 120 °C; black squares ☐: 7 d 120 °C.

The contents of the additives DEHP and 6PPD and the fuel ReadiJet, which are present in the elastomer in dependency on the sorption time, are reported in Figure 11A. DEHP and 6PPD are quantified with the external calibration, and the absolute contents are related to the filled elastomer mass $m_{0}$ without any further soluble additives. The overall mass change is determined by weighing the sample before and after the immersion in the fuel. It is evident that the changes in the sample mass during the sorption process is caused by the uptake of fuel into the elastomer and simultaneous desorption of the additives DEHP and 6PPD into the fuel which surrounds the NBR sample. Since the overall mass change is a convolution of both processes, it is corrected to only yield the mass uptake of ReadiJet by subtracting the known initial contents of DEHP and 6PPD from the initial elastomer mass: $m_{0}=m_{\mathrm{el}\;+\;\mathrm{additives}}\cdot (1-w_{\mathrm{DEHP},0}-w_{6\mathrm{PPD},0)}$. Details of this method are shown by Blivernitz et al. [46,47].

As described previously, the aromatics content of the absorbed liquid phase in the elastomer was quantified, as one can see in Figure 11B. The graph shows that the relative aromatics content in the equilibrium is higher than in the surrounding fuel. Whereas the neat ReadiJet contains 21.2 vol% aromatics, the liquid phase in the pristine sample contains 34.4 vol% aromatics at equilibrium. This is due to the higher affinity of aromatics towards NBR, which originates in interactions between the nitrile group of NBR and aromatic electrons [49]. At the beginning of the sorption (36.6 vol%), there is even a higher enrichment of the aromatics. When the elastomer is pre-swollen by aromatics, the other substances also enter the elastomer more easily so that there is a relative decrease in the aromatics content towards the equilibrium. The aromatics content also changes with proceeding sorption time and is different for pristine (34.4 vol%) and aged samples (39.1 vol%). With increasing aging time, the crosslinking and polarity of NBR is increased, making it harder for the fuel to enter the elastomer. Therefore, when the swelling is inhibited, the aromatic hydrocarbons are more enriched due to their high affinity toward NBR.

### 3.4. Intermediate Conclusions and Remarks

The properties of NBR deteriorate when the elastomer ages, which is expressed through its increased hardness and density as well as its decreased elongation and stress at break. The interaction of the elastomer with the synthetic fuel dynamically leads to a further change in its properties in dependency on the absorbed fuel. For aged elastomers, the fuel uptake is reduced. Gas chromatography coupled with mass spectrometry (GC/MS) adds valuable chemical information to the mechanical testing results. It allows one to separate the overall mass change into the uptake of fuel and the desorption of additives. Furthermore, it is possible to analyze the composition of the absorbed liquid in the elastomer with respect to its chemical nature. In the near future, a significant rise in the demand of synthetic fuels is expected, which makes this topic interesting to investigate experimentally.

## 4. Conclusions and Outlook

The swelling behavior of soft polymers is of great interest in the wide applications of elastomeric materials. The presented studies investigates the water, the fuel, and the fuel component toluene uptake using versatile testing methods. The first study highlights the pronounced swelling capability of several state-of-the-art additively manufactured materials depending on the solvent. As expected, the absorption of toluene is considerably more pronounced and reaches double the original weight (>100% mass gain). The absorption of water, on the other hand, reaches <5% but is of just as much interest, since water is omnipresent due to humidity and directly influences the printing result. For the additive manufacturing process, a concrete comparison between dry-printed material and wet-printed material is presented. Furthermore, the aspect of thermo-oxidative aging is investigated while the swelling behavior of pristine and aged elastomers in synthetic fuel ReadiJet is quantified. The characterization includes new experimental strategies such as a combination of sorption experiments with gas chromatography and mass spectrometry to precisely analyze the diffusion process from the surrounding liquid in and additives out of the elastomer. The quantified change in density and volume, the change in Shore hardness and the change in mechanical properties (elongation and stress at break) via tensile test highlight their need for experimental investigation.

## Figures and Tables

**Table 1:** Properties of investigated materials provided by the manufacturers.

Property	SILASTIC	Filaflex 70 A	Tango+ 70	Tango+ 50
Hardness [Shore A]	50	70	70	50
Tensile Strength [MPa]	9.5	32	3.5–5.0	1.9–3.0
Elongation at Break [%]	480	900	65–80	95–110
Printing Technology	LAM	FFF	MJ	MJ

**Table 2:** Ultimate stress and strain at break before and after water absorption.

Condition	Property	SILASTIC	Filaflex 70 A	Tango+ 70	Tango+ 50
Before water uptake	Stress [MPa]	11.69 ± 1.46	15.82 ± 1.62	3.73 ± 0.09	1.94 ± 0.02
	Strain [%]	674.47 ± 59.10	575.02 ± 16.57	78.23 ± 0.50	105.93 ± 0.71
After water uptake	Stress [MPa]	10.28 ± 1.23	14.57 ± 1.41	1.84 ± 0.02	1.35 ± 0.04
	Strain [%]	636.61 ± 51.35	630.99 ± 26.93	69.05 ± 0.28	97.27 ± 3.70

**Table 3:** Composition of the investigated NBR18 elastomer.

Component	Content/phr
Perbunan 1846	100
Di(2-ethylhexyl) phthalate (DEHP)	20
*N*-(1,3-dimethylbutyl)-*N*′-phenyl-*p*-phenylenediamine (6-PPD)	2
Carbon black (type: N550)	60
Zinc oxide	5
Stearic acid	1
Sulfur	2
*N*-Cyclohexyl-2-benzothiazole sulfenamide (CBS)	1.5
TMTM-80 ${}^{1}$	0.5

${}^{1}$ 80% tetramethylthiuram monosulfide, 20% elastomer binder and dispersing agents.

## Data Availability

Not applicable.

## Abbreviations

The following abbreviations are used in this manuscript:

6PPD	Antioxidant *N*-(1,3-dimethylbutyl)-*N*′-phenyl-*p*-phenylenediamine
AM	Additive Manufacturing
ARA	Applied Research Associates
ATR	Attenuated total Reflectance
CtL	Coal-to-Liquid
DEHP	Plasticizer Di(2-ethylhexyl)phthalate
FDM	Fused Deposition Modeling
FFF	Fused Filament Fabrication
FTIR	Fourier Transform Infrared Spectrometer
GC/MS	Gas Chromatography coupled with Mass Spectrometry
IR	Infrared
IATA	International Air Transport Agency
LAM	Liquid Additive Manufacturing
LSR	Liquid Silicone Rubber
MJ	Material Jetting
NBR	Acrylonitrile-butadiene-rubber
TPE	Thermoplastic Elastomer
TPU	Thermoplastic Polyurethane
